# Nutritional and Metabolic Characteristics of UK Adult Phenylketonuria Patients with Varying Dietary Adherence

**DOI:** 10.3390/nu11102459

**Published:** 2019-10-14

**Authors:** Benjamin Green, Robert Browne, Sarah Firman, Melanie Hill, Yusof Rahman, Kit Kaalund Hansen, Sarah Adam, Rachel Skeath, Paula Hallam, Ide Herlihy, Fiona Jenkinson, Claire Nicol, Sandra Adams, Lisa Gaff, Sarah Donald, Charlotte Dawson, Louise Robertson, Carla Fitzachary, Heidi Chan, Arlene Slabbert, Carolyn Dunlop, Alison Cozens, Camille Newby, Victoria Bittle, Gary Hubbard, Rebecca Stratton

**Affiliations:** 1 Medical Affairs, Nutricia Advanced Medical Nutrition, Wiltshire BA14 0XQ, UK; robert.browne@nutricia.com (R.B.); Heidi.chan@nutricia.com (H.C.); gary.hubbard@nutricia.com (G.H.); rebecca.stratton@nutricia.com (R.S.); 2 Guy’s and St Thomas’ NHS Foundation Trust, London SE1 9RT, UK; Sarah.Firman@gstt.nhs.uk (S.F.); Yusof.Rahman@health.nsw.gov.au (Y.R.); Carla.Fitzachary@gstt.nhs.uk (C.F.); Arlene.Slabbert@gstt.nhs.uk (A.S.); 3 Sheffield Teaching Hospitals NHS Foundation Trust, Sheffield S10 2JF, UK; Melanie.Hill@sth.nhs.uk; 4 University College London Hospitals NHS Foundation Trust, London WC1N 3BG, UK; kit.kaalundhansen@nhs.net; 5 Royal Hospital for Children, Glasgow G51 4TF, UK; Sarah.Adam@ggc.scot.nhs.uk; 6 Great Ormond Street Hospital for Children NHS Foundation Trust, London WC1N 3JH, UK; Rachel.Skeath@gosh.nhs.uk (R.S.); paula@tinytotsnutrition.co.uk (P.H.); Ide.Herlihy@gosh.nhs.uk (I.H.); 7 Royal Victoria Infirmary, Newcastle upon Tyne NE1 4LP, UK; Fiona.Jenkinson@nuth.nhs.uk (F.J.); Claire.Nicol@ggc.scot.nhs.uk (C.N.); Sandra.Adams@nuth.nhs.uk (S.A.); 8 Cambridge University Hospitals NHS Foundation Trust, Cambridge CB2 0QQ, UK; lisa.gaff@addenbrookes.nhs.uk (L.G.); sarah.donald@addenbrookes.nhs.uk (S.D.); 9 Queen Elizabeth Hospital, Birmingham B15 2TH, UK; Charlotte.Dawson@uhb.nhs.uk (C.D.); Louise.Robertson@uhb.nhs.uk (L.R.); 10 Royal Hospital for Sick Children, Edinburgh EH9 1LF, UK; Carolyn.Dunlop@nhslothian.scot.nhs.uk (C.D.); alison.cozens@nhs.net (A.C.); 11 Bristol University Hospitals NHS Foundation Trust, Bristol BS1 3NU, UK; Camille.Newby@UHBristol.nhs.uk (C.N.); Victoria.Bittle@UHBristol.nhs.uk (V.B.); 12 Faculty of Medicine, University of Southampton, Southampton SO16 6YD, UK

**Keywords:** phenylketonuria, PKU, adherence, nonadherence, nutrient intake, phenylalanine

## Abstract

The nutritional and metabolic characteristics of adult phenylketonuria (PKU) patients in the UK with varying dietary adherence is unknown. In other countries, nutritional and metabolic abnormalities have been reported in nonadherent patients compared to adherent counterparts. A pooled analysis of primary baseline data from two UK multi-centre studies was therefore performed to establish whether this is true from a UK perspective. Adult PKU patients who had provided 3-day food records and amino acid blood samples were included and grouped according to dietary adherence (adherent; *n* = 16 vs. nonadherent; *n* = 14). Nonadherent patients consumed greater amounts of natural protein compared to adherent patients (61.6 ± 30.7 vs. 18.3 ± 7.7 g/day; *q <* 0.001). In contrast, the contribution of protein substitutes to total protein intake was lower in nonadherent compared to adherent patients (3.9 ± 9.2 g/day vs. 58.6 ± 10.2 g/day; *q* < 0.001). Intakes of iron, zinc, vitamin D_3_, magnesium, calcium, selenium, iodine, vitamin C, vitamin A and copper were significantly lower in nonadherent compared to adherent patients and were below UK Reference Nutrient Intakes. Similarly, intakes of thiamin, riboflavin, niacin, vitamin B_6_ and phosphorus were significantly lower in nonadherent compared to adherent patients but met the UK Reference Nutrient Intakes. Phenylalanine concentrations in nonadherent patients were significantly higher than adherent patients (861 ± 348 vs. 464 ± 196 µmol/L; *q* = 0.040) and fell outside of European treatment target ranges. This study shows the nutritional and metabolic consequences of deviation from phenylalanine restriction and intake of PKU protein substitutes in nonadherent adult PKU patients. Collectively, these data further underlie the importance of life-long adherence to the PKU diet.

## 1. Introduction

Maintenance of blood phenylalanine concentrations within target range (120–600µmol/L) is recommended to avert the metabolic consequences of phenylketonuria (PKU; OMIM 261600). This is achieved through a careful, life-long, balance between phenylalanine restriction and intake of low- phenylalanine protein substitutes (herein referred to as the PKU diet). Mismanagement of this balance causes an excessive accumulation of circulating phenylalanine in blood, brain and body tissues [1]. The accumulation of phenylalanine saturates the transport system of amino acids into the brain (L-type amino acid transporter-1 (LAT-1)) and consequently becomes neurotoxic [1]. While a detailed description of the pathogenesis of phenylalanine neurotoxicity is beyond the scope of this manuscript, it likely manifests in a spectrum of progressive and sometimes irreversible neurological impairments including anxiety, depression, neuropsychological and executive functioning deficits [2]. 

Characterised by the avoidance of many protein-containing foods, the PKU diet is well established, safe and effective, yet severely restrictive. Low-phenylalanine protein substitutes are therefore integral adjuncts in the dietary management of PKU. They supply around 70–85% of patient’s protein requirements and the majority of essential and large neutral amino acids (in PKU, tyrosine is conditionally essential given the inability to perform phenylalanine hydroxylation effectively), optimise metabolic control and provide a major source of micronutrients [3]. Interestingly, large neutral amino acids have received increasing attention for their therapeutic potential to further improve outcomes in PKU [4]. Considering all large neutral amino acids share the same transport system to the brain, their presence in high concentrations competitively inhibits phenylalanine uptake at the gut-blood and blood-brain barrier [5]. Together, adherence to a phenylalanine restricted diet and intake of a protein substitute ensures a nutritionally adequate diet whilst enabling metabolic control.

Adherence with the PKU diet becomes increasingly challenging as patients’ age, especially as patients transition from adolescence to adulthood [2]. This, in part, may result from the poor organoleptic properties (taste, scent, texture) associated with the PKU diet and restriction of overall food choices [6]. Unfortunately, nonadherence with the PKU diet is widespread and commonly associated with disengagement from clinical review. In the US, 77% of adolescents and adults are believed to be nonadherent [7], with additional reports suggesting 52% (of 625 PKU-related respondents) find it difficult to follow the PKU diet [8]. These figures echo recent UK data, where 43% of adult patients report not following the PKU diet [9]. Moreover, reports suggest that 80% of UK and Australian adolescent and adult PKU patients have blood phenylalanine concentrations above target range [10].

Coupled with increased concentrations of circulating phenylalanine, nonadherent patients typically display eating habits which risk inadequate supply of many essential nutrients compared to their adherent counterparts [11,12]. Although data concerning micronutrient intake in patients with varying adherence is limited, the evidence available suggests vitamin B_12_, iron, zinc, vitamin D_3_, calcium, selenium, iodine [11,12] and long-chain polyunsaturated fatty acids are often inadequate in nonadherent patients yet sufficient in adherent counterparts [13,14]. Although evidence at present is inconclusive, PKU dietary nonadherence may also impact on the prevalence of overweight and obesity [15]. Whether the above observations stand true for patients in the UK is consequently unknown. We conducted a pooled analysis based on primary baseline data from two multi-centre studies to help further understand the nutritional and metabolic implications of PKU dietary nonadherence. 

## 2. Materials and Methods

### 2.1. Study Design and Ethics

A pooled analysis of primary baseline data from two multi-centre intervention studies (NCT03167697 and NCT02915510) was conducted in patients with PKU. Both studies were conducted in accordance with the Declaration of Helsinki of 1975, as revised in 2013, and ICH-Good Clinical Practice. The protocols were reviewed and approved by the Cambridge East 17/EE/0078 and London Central 16/LO/0543 research ethics committee respectively, where the methodological approach to dietary intake and amino acid analysis was identical between studies.

### 2.2. Recruitment, Inclusion Criteria, Exclusion Criteria and Study Population

#### 2.2.1. Recruitment

Male or female PKU patients (≥ 16 years) who were recruited to NCT03167697 and NCT02915510 between July 2016 and March 2019 were included in this study. All patients provided written informed consent at the time of recruitment. 

#### 2.2.2. Inclusion Criteria; NCT02915510

Patients from the NCT02915510 study were classified as adherent and were eligible for inclusion if they had taken a minimum of 20g protein equivalent from a low-phenylalanine protein substitute per day for at least one month prior to inclusion. 

#### 2.2.3. Inclusion Criteria; NCT03167697

Patients in NCT03167697 were classified as nonadherent and were eligible for inclusion if they had taken a maximum of 20g protein equivalent from a low-phenylalanine protein substitute per day for at least one month prior to inclusion and displayed blood phenylalanine concentrations of ≥ 600µmol/L.

#### 2.2.4. Exclusion Criteria

Patients were excluded across both studies if they were currently prescribed sapropterin or a similar tetrahydrobiopterin-based medication, were pregnant or lactating, required parenteral nutrition, had major hepatic or renal dysfunction or had participated in other studies within 1 month.

#### 2.2.5. Study Population

In total, this study comprised of 30 (≥ 16 years) free-living community-based adult patients with PKU. Demographics (age, gender), anthropometric measures (weight, height and body mass index), and historical blood phenylalanine were recorded at the time of recruitment.

### 2.3. Dietary and Nutritional Intake

Baseline dietary intake was evaluated over 3 consecutive days via food records. Patients provided comprehensive recordings of all food, drink and protein substitute(s) consumed, providing weights or portion sizes of each food item to allow for analysis. Methods of preparation and cooking, and names of branded products were also requested. For homemade dishes, patients were asked to record individual ingredients and quantities for the whole dish, along with a brief description of cooking method and how much of the dish they consumed. Nutritional analysis was performed using the software package Nutritics (Nutritics Research Edition v5.042, Dublin, Ireland).

Food records were analysed for energy, macro and micronutrients with particular focus on vitamin B_12_, iron, zinc, vitamin D_3_, magnesium, calcium, selenium and iodine as these are characteristically low in a nonadherent PKU population [11,12]. Intakes of micronutrients were compared against the UK Reference Nutrient Intakes (RNI) to determine nutritional adequacy [16].

### 2.4. Amino Acid Profile

In addition to collecting historical blood phenylalanine information, blood samples for proteinogenic amino acids, non-proteinogenic amino acids and 2-aminoethanesulfonic acid analysis were collected via dried blood spot following an overnight fast by fingertip puncture. Once collected, samples were sent to an independent third-party accredited laboratory for analysis (Genova Diagnostics Europe, CPA number 3054). Amino acids were quantified from whole blood via high performance liquid chromatography (2695 HPLC Separations Module, Waters). The intra-assay coefficient of variation reported by the manufacturer was < 10% for all amino acids and for phenylalanine was < 8%. The use of dried blood spot for amino acid analysis offers practical and analytical advantages [17] and is a universally accepted method in current clinical practice in the UK.

### 2.5. Statistical Analysis

Statistical procedures were performed using software package IBM SPSS Statistics v24 IBM SPSS v24.0, Armonk, NY, USA). Data were checked for normal distribution with the use of the Kolmogorov-Smirnov normality test and were log-transformed if appropriate before statistical analysis. Extreme outliers, defined as results falling >3 interquartile ranges outside the 1st or 3rd quartiles for the combined study group, were removed. Statistical analysis comprised of independent *t*-tests for comparison between groups. For patient characteristics and phenylalanine to tyrosine ratio, statistical significance was accepted at an α level of *p* < 0.05. The *p* values for nutritional and blood amino acid analysis were corrected using the Benjamini-Hocheberg method, to control the false discovery rate [18]. Corrected *p* values (reported as *q* values in the text and tables) were calculated using an acceptable false discovery rate of 5%, where *q* values of < 0.05 were considered statistically significant. All data are presented as mean ± SD unless otherwise stated.

## 3. Results

Characteristics of the 30 adult PKU patients included in this study are provided in Table 1, with *n* = 16 in the adherent group and *n* = 14 in the nonadherent group. Age and proportion of males:females were similar between groups. All patients in the adherent group were following a phenylalanine restricted diet and were reported to be generally very adherent in taking their prescribed amount of low-phenylalanine protein substitute. The amounts of low-phenylalanine protein substitutes prescribed in this group (in grams of protein equivalent per day) included 40g/day (*n* = 2), 60 g/day (*n* = 12), 70g/day (*n* = 1) and 80g/day (*n* = 1). The nonadherent patients had not been following the PKU diet for an average of 5.4 years (range 1–18 years). From the *n* = 14 patients in the nonadherent group, *n* = 2 were following a relaxed PKU diet (taking 20g protein equivalent from a low-phenylalanine protein substitute but not restricting natural protein), *n* = 1 were following a low protein diet but taking no low-phenylalanine protein substitutes and *n* = 11 patients were following an unrestricted diet and consuming no low-phenylalanine protein substitutes. Mean body weight, body mass index and historical blood phenylalanine were significantly higher in the nonadherent group, as compared to the adherent group. 

### 3.1. Dietary and Nutritional Intake

#### 3.1.1. Energy and Macronutrient Intakes

**Figure 1:**
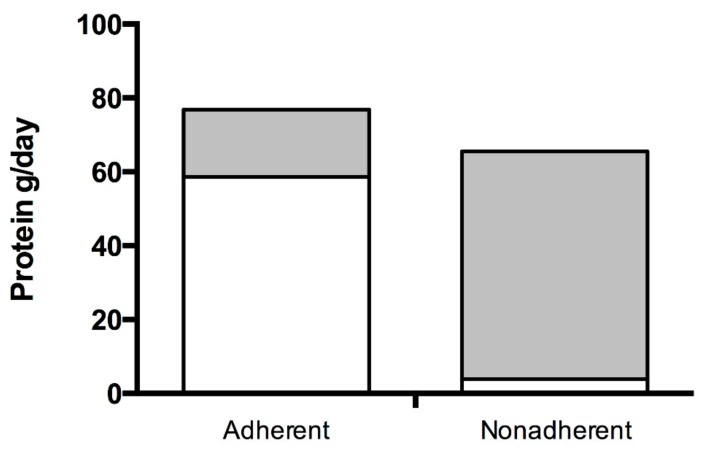
Contribution of low-phenylalanine protein substitutes (expressed as protein equivalents) and natural protein to total protein intake of adherent and nonadherent patients. White shaded bars [⬜] represent protein equivalents from low-phenylalanine protein substitutes whereas grey shaded bars [⬛] represent natural protein intake.

Daily energy (adherent: 1813 ± 445 kcal/day vs. nonadherent: 1609 ± 426 kcal/day; *q* = 0.240), total protein (adherent: 76.9 ± 13.4 g/day vs. nonadherent: 65.5 ± 27.2 g/day; *q* = 0.168, Figure 1) and percentage of energy derived from protein (adherent: 17.7 ± 4.12%EN vs. nonadherent: 16.0 ± 5.2%EN; *q* = 0.341) were similar between groups. Contribution of low-phenylalanine protein substitutes to total protein intake, however, was greater in adherent compared to nonadherent patients (58.6 ± 10.2 g/day vs. 3.9 ± 9.2 g/day, respectively; *q* < 0.001, Figure 1). Conversely, the contribution of natural protein to total protein intake was lower in adherent compared to nonadherent patients (18.3 ± 7.7 g/day vs. 61.6 ± 30.7 g/day, respectively; *q <* 0.001, Figure 1). 

Carbohydrate intake was greater in adherent compared to nonadherent patients (260 ± 83.0 g/day vs. 190 ± 63.1 g/day, respectively; *q* = 0.046), whereas the opposite was apparent for total fat intake (adherent: 49.0 ± 13.9 g/day vs. nonadherent: 61.4 ± 16.3 g/day) although this was not significant after Benjamini-Hocheberg adjustment (*q* = 0.065). Compared to nonadherent patients, adherent patients consumed a higher proportion of energy from carbohydrate (adherent: 56.5 ± 5.94 vs nonadherent: 46.8 ± 7.28%EN of total energy intake; *q* = 0.001) and a lower proportion of energy from fat (adherent: 24.6 ± 4.77 vs. nonadherent: 35.4 ± 8.19 %EN of total energy intake; *q* < 0.001). 

#### 3.1.2. Micronutrient Intakes

**Figure 2:**
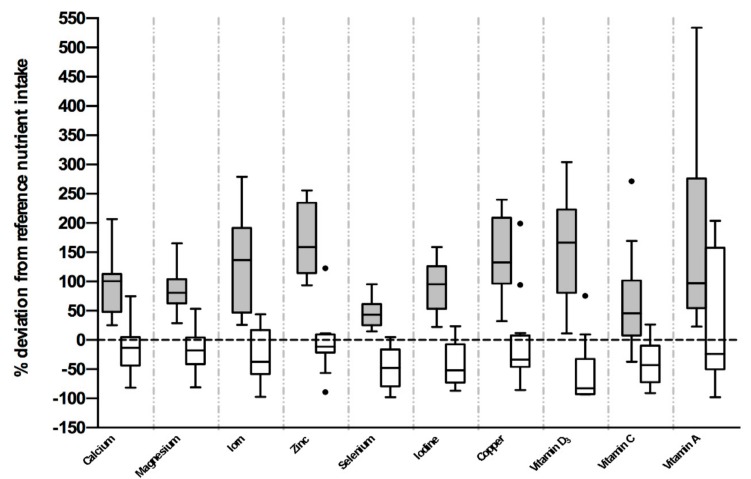
Nutrient intake is depicted as percent deviation from age and gender specific UK reference nutrient intakes. Tukey box and whisker plot. Grey shaded boxes [⬛] represent the adherent patients, whereas white shaded boxes [⬜] represent values obtained from the nonadherent population.

Intakes of iron, zinc, vitamin D3, magnesium, calcium, selenium, iodine, vitamin C, vitamin A, copper, thiamin, riboflavin, niacin, vitamin B_6_, biotin, pantothenic acid, vitamin E and vitamin K_1_ were significantly lower in nonadherent compared to adherent patients (Table 2). Intakes of manganese, potassium, vitamin B_12_, sodium, chloride and folate were similar between groups. In adherent patients, mean and median intakes of iron, zinc, vitamin D_3_, magnesium, calcium, selenium, iodine, vitamin C, vitamin A, copper, thiamin, riboflavin, niacin, and vitamin B_6_ met the UK Reference Nutrient Intake. In nonadherent patients, mean and median intakes of iron, zinc, vitamin D_3_, magnesium, calcium, selenium, iodine, vitamin C, copper were below the UK RNI but were met for thiamin, riboflavin, niacin, vitamin A, phosphorus, vitamin B_6_, sodium, chloride and folate. Percentage deviation of iron, zinc, vitamin D_3_, magnesium, calcium, selenium, iodine, vitamin C, vitamin A and copper from UK RNI are depicted in Figure 2.

With the exception of vitamin D_3_ and copper (as no UK Lower Reference Nutrient Intakes (LRNI) are available), all patients in the adherent group met the UK LRNI for all micronutrients assessed (except for potassium). Some nonadherent patients failed to meet the UK LRNI for iron, zinc, magnesium, calcium, selenium, iodine, vitamin A and vitamin B_12_. For selenium and iodine this was true for 50% (*n* = 6 of 12) of nonadherent patients. For calcium and magnesium this was true for 25% (*n* = 3 of 12) of nonadherent patients and for iron, zinc and vitamin B_12_ this was true for 42% (*n* = 5 of 12), 18% (*n* = 2 of 12) and 18% (*n* = 2 of 12), respectively. Although the UK RNI were met for riboflavin, niacin, vitamin A and folate, some nonadherent patients still failed to meet the UK LRNI for these micronutrients. For riboflavin this was true for 18% (*n* = 2 of 12) of nonadherent patients and for 8% (*n* = 1 of 12) for niacin, folate and vitamin A, respectively. 

### 3.2. Amino Acid Profile

All amino acids were within normal range of the general population in both groups except for phenylalanine. Phenylalanine concentrations for patients in the adherent group were within European treatment target ranges [18]. Phenylalanine concentrations for nonadherent patients were significantly higher than adherent patients and fell outside of treatment target ranges (Table 3). Concentrations of tryptophan were also significantly higher in adherent compared to nonadherent patients (Table 3). Methionine and threonine were initially significantly higher in adherent patients compared to nonadherent patients, although were not significant after Benjamini-Hocheberg adjustment (Table 3). All other amino acids were similar between groups. Phenylalanine to tyrosine ratio was significantly higher in nonadherent compared to adherent patients (17.9 ± 6.92 vs. 9.60 ± 4.25, *p* = 0.001). 

## 4. Discussion

Dietary management in PKU through phenylalanine restriction and intake of low-phenylalanine protein substitutes is needed to optimise metabolic control while preventing protein and micronutrient deficiencies [3]. Adherence with the PKU diet, however, can become challenging for some patients as they transition from adolescence to adulthood [2] and it is increasingly evident that PKU dietary nonadherence is associated with nutritional and metabolic abnormalities. We believe this is the first comparative study to examine nutritional and metabolic characteristics of adherent and nonadherent adults with PKU in the UK. To the best of our knowledge it also includes the largest number of nonadherent patients as compared to other studies [11,12]. The results presented here support previous observations and further confirm that nonadherent adult PKU patients have an insufficient intake of key micronutrients and poor metabolic (blood phenylalanine) control compared to adherent counterparts [11,12].

In this study, nonadherent patients displayed a significantly higher body mass index compared to their adherent counterparts, corroborating earlier findings [20]. Although evidence at present is inconclusive, nonadherence to the PKU diet may impact on the prevalence of overweight and obesity [15], which over time may have implications for increased cardiovascular risk compared to compliant patients [21]. It is unknown if this is a consequence of the underlying condition, treatment and/or dietary choices, or an outcome of inadequate metabolic control. While avoidance of excess energy intake is key to protect against overweight and obesity, in this study daily energy intake was similar between groups, although low, which may suggest some patients underreported dietary intakes. Carbohydrate intakes (total intakes and percentage contribution to total energy intake) were greater in adherent compared to nonadherent patients, whereas the opposite was apparent for fat intakes and supports earlier findings [11]. In an adherent PKU population the percentage contribution of carbohydrate to total energy intake is typically greater (59 to 67% energy) than recommendations for a general population [14], and fat intakes are typically lower (20–25% energy) [22], largely due to the avoidance of fat/protein containing foods (e.g., dairy or meat) [22]. This was shown for the adherent patients in this study, however, the percentage contribution of fat to total energy intake for nonadherent patients was 35.4% and can be attributed to a higher consumption of natural/animal protein and processed convenience foods (e.g., chips, pizzas, crisps and takeaways). Together, natural protein (65.5 ± 27.2 g/day) and fat intakes of nonadherent patients reflect intakes of the general population of the UK [23]. The intake of natural protein in the nonadherent patients was excessive and is likely responsible for the high phenylalanine concentrations observed in this study. Although we did not quantify it in this study, it is conceivable that the nonadherent patients had greater phenylalanine uptake in the brain and inhibited neurotransmitter, catecholamine and hormone production.

While a phenylalanine-restricted diet presents many potential nutritional shortfalls, intake of low-phenylalanine protein substitutes represents the major source of micronutrients necessary to combat the risk of nutritional insufficiency [24] which is clearly evidenced throughout this study. The data from a nonadherent perspective provide further evidence of unfavourable eating behaviours in PKU with deviation from phenylalanine restriction and intake of PKU protein substitutes. In this sense, despite compensatory increases in natural protein intake, nonadherent patients had significantly lower intakes of iron, zinc, vitamin D_3_, magnesium, calcium, selenium, iodine, vitamin C, vitamin A and copper compared to adherent patients and intakes of these nutrients were also below UK RNI for all. These observations are most likely explained due to the lack of low-phenylalanine protein substitute intake in nonadherent patients and may also represent consequences of poor diet quality compared to adherent counterparts. For iron, zinc, vitamin D_3_, magnesium, calcium, selenium and iodine this again supports earlier findings from Switzerland and Germany [11,12]. Significantly lower intakes of thiamin, riboflavin, niacin, vitamin B_6_ and phosphorus were also reported in nonadherent compared to adherent patients but met the UK RNI, which to our knowledge has not previously been reported in literature concerning adult PKU patients with varying adherence. The long-term consequences of these differences are unknown, but efforts to establish any long-term implications are encouraged. Of concern, a proportion of nonadherent patients failed to meet the LRNI for the micronutrients measured. Nonadherence could therefore have implications on bone health [25]. Few studies report suboptimal bone health in PKU which may be explained by inadequate intakes of calcium and vitamin D_3_ [26] but could also be influenced by raised phenylalanine concentrations as a consequence of nonadherence [20]. With nonadherence, evidence also shows impairments in executive functioning, information processing (reaction times, attention) and mood (increased inhibition, anxiety, depression and low self-esteem) have also been reported in nonadherent PKU patients and likely transpire due to the pathophysiological consequences of disrupted phenylalanine hydroxylation [27,28].

Notwithstanding the differences in blood phenylalanine concentrations, we observed significantly greater concentrations of tryptophan in adherent compared to nonadherent patients. Methionine and threonine were initially significantly higher in adherent patients compared to nonadherent patients, although were not significant after Benjamini-Hocheberg adjustment. While absolute values were within the 95% reference range for both groups, the differences observed may be unsurprising considering methionine, threonine and tryptophan are essential amino acids and are typically found in high biological value foods, which are often lacking from a nonadherent PKU die t [8]. In addition, these amino acids are commonly added to PKU protein substitutes in enhanced quantities. Large neutral amino acids, such as methionine, threonine and tryptophan provided in PKU protein substitutes compete with phenylalanine for LAT-1 transport at a gut and blood-brain barrier level [29,30]. Although there is limited information concerning the bioavailability of amino acids in PKU protein substitutes, it could be argued that adherent patients consumed a greater amount of methionine, threonine and tryptophan via low-phenylalanine protein substitutes which was to some extent reflected in their blood results as previously suggested [31].

Though the work presented throughout this manuscript has numerous strengths, the findings of this pooled analysis are not without limitation. Firstly, it is relevant to acknowledge that the results of this study are limited to a relatively small population of adult PKU patients which may warrant care when interpreting the conclusions. Although this study included the largest number of nonadherent patients compared to earlier studies, which is an achievement worth acknowledgement, a purposefully designed and powered study with blood micronutrients and an increased number of adherent and nonadherent patients would be of benefit to strengthen conclusions. It would also be worthwhile if groups were of equal sizes. Secondly, accurately quantifying nutritional intake is difficult, particularly in free-living settings. Prospective approaches such as self-reported food records are acknowledged as the gold-standard approach to assess energy intake and feeding behavior [32], yet present opportunities for bias and misreporting. While self-reported food records have not yet been validated against an external criterion (e.g. doubly labelled water) in PKU and the validity, precision and accuracy of this technique over differing data collection periods is consequently unknown, one could argue a 3-day food record is not the best representation of habitual dietary behaviours compared to 7-day food records, which are accepted as the gold-standard approach. It also makes it difficult to establish the likely level of underreporting. In this study, it is probable that underreporting occurred in both groups and the nutritional differences reported here may be a product of underreporting. Moreover, underreporting bias of the food records may be potentially influenced by weight status between groups as is sometimes seen in dietary evaluation studies, yet this cannot be concretely confirmed without further study. When working with rare disease populations, however, it is important to adopt methods that are non-invasive and exert a low level of participant burden. Self-reported weighed food diaries elicit considerable burden and it was for this reason we opted for 3-day self-reported food records. Patients are required to meticulously weigh and document all food and drink items to track natural protein and phenylalanine intake daily as part of their care. The approach to assess nutritional intakes in this study may therefore represent a valid approach to quantify habitual dietary behaviours in PKU as the technique is familiar to patients [33], but the nutrient intake data should certainly be extrapolated with caution. 

## 5. Conclusions

This study shows that in nonadherent UK adult PKU patients, deviation from phenylalanine restriction and intake of low-phenylalanine protein substitutes leads to lower micronutrient intake and poor metabolic control. These data confirm earlier observations from other countries and illustrate similar nutritional and metabolic consequences with PKU dietary nonadherence in the UK. The results of this study may therefore have wider application and could be used globally to further underlie the importance of life-long adherence with the PKU diet. Collectively, these data underlie the importance of life-long adherence with the PKU diet. Reinstating a strict PKU diet may resolve executive functioning, information processing and mood complications, while restoring metabolic control and nutritional adequacy. While simple in theory, research demonstrates the return to strict adherence is extremely difficult [34], with many patients again nonadherent after several months. Based on recent data, new methods are emerging that can be effectively used to re-engage nonadherent PKU patients in their dietary management through a low burden regimen with immediate nutritional and possible mood benefits [35]. A stepwise approach with the overarching aim of achieving full dietary adherence over a set time may therefore be a more appropriate approach to facilitate patients’ return to the PKU diet but should be confirmed with further research. Nonetheless, the results of this study should stimulate conversations between metabolic practitioners and patients to continue voicing the importance of diet for life which hopefully improves rates of dietary adherence in adults with PKU in the UK. 

## Figures and Tables

**Table 1:** Patient characteristics.

	Adherent (*n* = 16)	Nonadherent (*n* = 14)	*p* Value
Age, y	29.5 ± 11.2	33.9 ± 8.5	0.240
Male: Female, n:n	7:9	5:9	0.654
Body weight, kg	71.1 ± 15.7	88.8 ± 20.8	0.017
Body mass index, kg/m^2^	24.9 ± 3.8	31.8 ± 7.6	0.006
Historical Phenylalanine, µmol/L ^a^	618 ± 292 ^b^	1050 ± 341	0.001

^a^ Mean blood phenylalanine concentration from past 3 historical blood phenylalanine tests prior to recruitment. ^b^ For some patients, at the time of recruitment, UK target ranges for adult patients was 120–700µmol/L (EU-wide target ranges updated to 120–600µmol/L in January 2017).

**Table 2:** Micronutrient Intakes in Adherent and Nonadherent patients.

		Adherent (*n* = 15) ^a^	Nonadherent (*n* = 12) ^b^	Unadjusted *p* Value	Adjusted *q* Value
**Minerals**					
Sodium	mg	1673 ± 574	1958 ± 834	0.304	0.330
Potassium	mg	2983 ± 1152	2305± 949	0.113	0.135
Chloride	mg	2955 ± 2153	2777 ± 1089	0.681	0.709
Calcium	mg	1489 ± 337	619 ± 319	<0.001	<0.001
Phosphorus	mg	1456 ± 332	955 ± 415	0.002	0.003
Magnesium	mg	521 ± 123	238 ± 98.7	<0.001	<0.001
Iron	mg	26.9 ± 5.61	8.61 ± 5.02	<0.001	<0.001
Zinc	mg	22.3 ± 5.52	7.26 ± 3.53	<0.001	<0.001
Copper	µg	2.99 ± 0.70	1.14 ± 0.94	0.003	0.004
Manganese	mg	4.97 ± 1.55	4.47 ± 5.75	0.081	0.101
Selenium	µg	95.8 ± 16.4	34.4 ± 21.9	<0.001	<0.001
Iodine	µg	266 ± 57.0	80.5 ± 50.2	<0.001	<0.001
**Vitamins**					
Vitamin A	µg RE	1842 ± 1138	1162 ± 1494	0.018	0.025
Vitamin D_3_	µg	25.8 ± 8.96	4.16 ± 5.31	<0.001	<0.001
Vitamin E	mg α-TE	21.5 ± 5.71	7.6 ± 4.88	0.001	0.001
Vitamin K_1_	µg	122 ± 38.1	34.2 ± 35.6	<0.001	0.001
Thiamin	mg	2.82 ± 0.77	1.37 ± 0.81	0.014	0.020
Riboflavin	mg	2.90 ± 0.58	1.29 ± 0.57	0.001	<0.001
Niacin	mg	58.7 ± 12.9	31.1 ± 16.5	<0.001	<0.001
Pantothenic acid	mg	10.4 ± 2.24	4.64 ± 2.10	<0.001	<0.001
Vitamin B_6_	mg	3.55 ± 0.79	1.93 ± 1.10	0.019	0.025
Folate	µg	203 ± 134	231 ± 115	0.845	0.845
Vitamin B_12_	µg	5.70 ± 1.19	4.18 ± 3.97	0.223	0.253
Biotin	µg	196 ± 38.8	34.8 ± 35.2	<0.001	<0.001
Vitamin C	mg	229 ± 122	89.4 ± 65.3	0.001	0.003

^a^ For the adherent group, *n* = 1 patient provided unusable diet data. ^b^ For the nonadherent group, *n* = 2 patients did not provide diet data. An additional *n* = 1 patient was excluded from vitamin B_12_ analyses as the result was an extreme outlier (>3 IQR above 3rd quartile).

**Table 3:** Blood Amino Acid Profile in Adherent and Nonadherent patients.

	95% Reference Range µmol/L ^a^	Adherent (*n* = 16)	Nonadherent (*n* = 13)	Unadjusted *p* Value	Adjusted *q* Value
Arginine	17–91	41.3 ± 12.7	40.2 ± 16.2	0.848	0.923
Asparagine	42–88	49.6 ± 9.54	51.0 ± 7.35	0.673	1.121
Aspartic Acid	26–233	112 ± 37.3	144 ± 51.7	0.225	0.643
Citrulline	16–51	28.8 ± 12.6	26.5 ± 6.81	0.651	1.184
Glutamic acid	97–258	155 ± 84.2	178 ± 45.7	0.127	0.423
Glutamine	209–573	306 ± 81.7	302 ± 102	0.922	0.922
Glycine	207–559	344 ± 78.3	325 ± 90.7	0.556	1.112
Histidine	22–99	67.9 ± 24.8	74.4 ± 16.5	0.429	0.953
Isoleucine	28–96	45.3 ± 16.6	46.2 ± 13.1	0.772	0.965
Leucine	59–162	89.0 ± 20.6	91.5 ± 19.6	0.746	1.066
Lysine	63–220	111 ± 27.9	104 ± 13.1	0.376	0.940
Methionine	10–33	19.2 ± 6.25	13.9 ± 3.23	0.025	0.167
Ornithine	50–210	104 ± 99.2	83.2 ± 19.0	0.777	0.914
**Phenylalanine**	120–600 ^b^	464 ± 196	861 ± 348	0.002	0.040
Serine	79–310	181 ± 112	171 ± 70.0	0.880	0.926
Taurine	124–282	172 ± 30.8	202 ± 49.5	0.053	0.212
Threonine	54–169	112 ± 44.8	82.8 ± 23.7	0.026	0.130
Tryptophan	24–52	36.6 ± 6.47	30.1 ± 4.27	0.004	0.040
Tyrosine	36–99	47.8 ± 11.2	49.3 ± 13.1	0.743	1.143
Valine	105–266	162 ± 39.2	156 ± 26.9	0.764	1.019

^a^ 95% reference ranges for the measured amino acids in a healthy population as provided by Genova Diagnostics. ^b^ European treatment target ranges for phenylalanine for non-pregnant individuals older than 12 years [19].
